# Coping With Changes to Sex and Intimacy After a Diagnosis of Metastatic Breast Cancer: Results From a Qualitative Investigation With Patients and Partners

**DOI:** 10.3389/fpsyg.2022.864893

**Published:** 2022-04-06

**Authors:** Jennifer Barsky Reese, Lauren A. Zimmaro, Sarah McIlhenny, Kristen Sorice, Laura S. Porter, Alexandra K. Zaleta, Mary B. Daly, Beth Cribb, Jessica R. Gorman

**Affiliations:** ^1^Cancer Prevention and Control Program, Fox Chase Cancer Center, Philadelphia, PA, United States; ^2^Department of Psychiatry & Behavioral Sciences, Duke University Medical Center, Durham, NC, United States; ^3^Research and Training Institute, Cancer Support Community, Philadelphia, PA, United States; ^4^Department of Clinical Genetics, Fox Chase Cancer Center, Philadelphia, PA, United States; ^5^Cancer Support Community of Greater Philadelphia, Philadelphia, PA, United States; ^6^College of Public Health and Human Sciences, Oregon State University, Corvallis, OR, United States

**Keywords:** metastatic breast cancer, couples therapy, interventions, qualitative research, sexuality

## Abstract

**Objective:**

Prior research examining sexual and intimacy concerns among metastatic breast cancer (MBC) patients and their intimate partners is limited. In this qualitative study, we explored MBC patients’ and partners’ experiences of sexual and intimacy-related changes and concerns, coping efforts, and information needs and intervention preferences, with a focus on identifying how the context of MBC shapes these experiences.

**Methods:**

We conducted 3 focus groups with partnered patients with MBC [*N* = 12; M age = 50.2; 92% White; 8% Black] and 6 interviews with intimate partners [*M* age = 47.3; 83% White; 17% Black]. Participants were recruited through the Fox Chase Cancer Center Tumor Registry and the Cancer Support Community. Qualitative data were analyzed using the Framework Method and Dedoose software.

**Results:**

Qualitative analyses revealed several key themes reflecting ways in which MBC shapes experiences of sex/intimacy: (1) the heavy disease/treatment burden leads to significant, long-term sexual concerns (e.g., loss of interest and vaginal dryness/discomfort) and consequent heightened emotional distress for both patients (e.g., guilt around not being able to engage in intercourse) and partners (e.g., guilt around pressuring the patient to engage in sexual activity despite pain/discomfort); (2) viewing the relationship as having “an expiration date” (due to expected earlier mortality) influences patients’ and partners’ concerns related to sex/intimacy and complicates coping efforts; and (3) information needs extend beyond managing sexual side effects to include emotional aspects of intimacy and the added strain of the life-limiting nature of the disease on the relationship. The heightened severity of sexual concerns faced by patients with MBC, compounded by the terminal nature of the disease, may place patients and partners at risk for significant adverse emotional and interpersonal consequences.

**Conclusion:**

Findings suggest unique ways in which sex and intimate relationships change after a diagnosis of metastatic breast cancer from both patients’ and partners’ perspectives. Consideration of the substantial physical and emotional burden of MBC and the broader context of the relationship and intimacy overall is important when developing a sexuality-focused intervention in this population. Addressing sexual concerns is a critical part of cancer care with important implications for patients’ health and quality of life.

## Introduction

With treatments continually improving for metastatic breast cancer (MBC), nearly one-third of women with this diagnosis can expect 5-year survival (Siegel et al., 2020). In light of these data, and clinical survivorship guidelines that apply to those living with chronic cancers (i.e., metastatic disease; National Comprehensive Cancer Network, 2021), there are growing calls to address cancer-related symptoms and side effects for patients with metastatic disease, including in their sexual function and intimate relationships (Di Lascio and Pagani, 2017; Langbaum and Smith, 2019). Contrary to common beliefs about patients with advanced cancer (e.g., that sex is not a priority), data suggest that patients with MBC report significant sexual concerns, wish to maintain physical intimacy in their relationships, and would like support in coping with treatment-related sexual concerns (Andersen, 2009; McClelland et al., 2015; McClelland, 2016; Reese et al., 2016).

Sexual concerns are reported by 50 to 80% of patients with a diagnosis of MBC (Reese et al., 2010b; Gambardella et al., 2018). Common sexual problems related to breast cancer treatments include those that are physical, including vaginal dryness and discomfort (Burwell et al., 2006; Alder et al., 2008), motivational/emotional, including decreased sexual interest (Fobair et al., 2006; Ochsenkuhn et al., 2011), and interpersonal in nature, including avoidance of sexual activity (Gilbert et al., 2010; Bredart et al., 2011; Loaring et al., 2015). Other common concerns include post-surgery breast changes that can influence body image and sexual activity, such as the loss of the breasts (Djohan et al., 2010; Otterburn et al., 2010). Because breast cancer-related sexual problems can be persistent (Raggio et al., 2014), it is critical to develop efficacious interventions to address these concerns.

In light of the lifespan limitations associated with having a terminal disease and the heightened physical and emotional symptom burden associated with metastatic disease and its treatments (Vilhauer, 2008; Mosher and DuHamel, 2012; Mosher et al., 2013), and prior research suggesting that women with MBC voice unique psychosocial impacts of their disease relative to those with early stage disease (Vilhauer, 2011), it seems likely that patients with MBC could have unique experiences related to the effects of their cancer on sex and intimacy. Yet, relatively few studies have examined changes to sex and intimacy after a diagnosis of MBC (Drageset et al., 2021), limiting our understanding of the sexual and intimacy-related concerns and experiences of this population (Silverman and Rabow, 2018). In addition, because partners of women with breast cancer also report sexual, relationship, and psychological problems associated with the cancer (Zahlis and Lewis, 2010; Milbury and Badr, 2012; Zimmermann, 2015), and because women generally involve their partners in coping with sexual concerns (Reese et al., 2016), it is essential to understand partners’ perspectives within this research. Yet, the bulk of the research examining sexual issues for women with advanced (e.g., recurrent) or metastatic breast cancer has included only patients (Andersen et al., 2007; Andersen, 2009; McClelland et al., 2015; McClelland, 2016), and these studies have been critical in identifying key issues in women’s experiences in the context of MBC, they have not shed light on partners’ perspectives on the same types of experiences. Alternatively, studies of psychosocial issues in MBC that have included partners, while highly informative, did not examine sexual issues (Badr et al., 2010; Milbury and Badr, 2012). Finally, of the psychosocial interventions aimed at addressing sexual concerns in patients with breast cancer, nearly all have excluded those with MBC (Schover et al., 2013; Candy et al., 2016; Hummel et al., 2017b; Reese et al., 2019, 2020b; Sopfe et al., 2021), making it difficult to know whether these interventions would apply to or be effective for patients living with MBC. In sum, there is a gap in information that could inform interventions addressing sexual concerns in this population, including how MBC patients and partners experience and cope with such concerns, as well as information needs and intervention preferences.

The objective of this study was thus to characterize the experiences of sexual and intimacy-related changes and concerns for patients and intimate partners facing an MBC diagnosis through a qualitative investigation with an eye toward intervention development for this population. The following primary research questions guided the present investigation: (1) What are the sexual and intimacy-related changes (i.e., physical, emotional, and relational) and concerns for patients with MBC and partners of patients with MBC?; (2) What are patients’ and partners’ experiences of coping with these concerns?; and (3) What are patients and partners’ information needs and intervention preferences? For all three questions, we were particularly interested in understanding how the context of metastatic disease may have shaped patients’ and partners’ experiences. We include both the terms “sexual” and “intimacy” in our aims and methods because we recognize that patients’ experiences of physical intimacy may or may not include sexual activity, *per se*, and aim for inclusivity and breadth in our investigation. For our purposes, we define intimacy as an interpersonal process involving mutual sharing and understanding and feelings of closeness, warmth, and affection, with both physical and emotional aspects (Reese et al., 2012).

## Materials and Methods

### Setting and Participant Eligibility

The study protocol and procedures were reviewed and approved by the Institutional Review Board at Fox Chase Cancer Center (Protocol #20–1,039). The consolidated criteria for reporting qualitative research (COREQ) checklist was followed in reporting the methods and findings of this study (Tong et al., 2007). Participants completed online consent prior to the focus group or interview. Women aged 18 years or older were eligible if they (a) had a diagnosis of metastatic breast cancer (i.e., Stage IV), (b) had a romantic partner, and (c) were willing to have their participation in the qualitative study audio-recorded. Partners of women meeting these criteria were also eligible to participate. There were no limitations in eligibility based on gender identity, sexual orientation, or marital status. Exclusion criteria included the inability to read or speak English, or having a poor functional status (i.e., capable of only limited self-care or being completely disabled, as determined by an Eastern Cooperative Oncology Group (ECOG) performance score > 2; Oken et al., 1982) or being medically unable to participate as judged by a physician, medical record, or self-report.

### Recruitment

In an attempt to diversify our sample, we recruited through both convenience methods (i.e., mailings to pre-screened patients identified using the Fox Chase Tumor Registry) and community-based stakeholder engagement methods (i.e., email, social media, and online community posts) in partnership with the Cancer Support Community, a national non-profit advocacy organization, and Cancer Support Community of Greater Philadelphia. A trained research assistant conducted eligibility screening privately over the phone with patients who were interested in participating. Interested patients were asked for permission to contact their partners about participation and for their contact information. Partners for whom this information was provided were then contacted and screened privately over the phone. If a potential participant screened as eligible, the research assistant discussed the study details and confidentiality and provided the opportunity for the potential participant to ask any questions and to enroll if interested. Participants who agreed to enroll then provided written consent and filled out an availability form to help facilitate scheduling. Participants received $40 in gift cards for their participation. Participants had no personal knowledge of the interviewers (JBR, LZ) or members of the research team prior to or during data collection that could potentially bias data.

### Data Collection

Data were collected in February–April 2021. All patient participants (*N* = 12) participated in one of three 90-min focus groups. Focus groups were selected because the shared nature of the conversations can often elicit deep discussion of issues, even for sensitive topics like sexuality; we have used this format in other qualitative studies on a similar topic in breast cancer (Reese et al., 2016, 2017). In past studies with a similar focus by our team (Reese et al., 2016), three focus groups provided sufficient information; we planned the number of focus groups based on this prior experience, with the understanding that if thematic saturation were not achieved we could conduct more. Partners were recruited for individual interviews (*N* = 6, 60- to 75-min interviews each) once it was determined that scheduling and recruitment challenges would render the focus group format unfeasible for study partners. All other aspects of the qualitative interviewing process were similar across the focus group and individual interview formats; no repeat interviews were carried out; All focus groups and interviews were conducted virtually using password protected Zoom meetings and led by female licensed psychologists who had at least severe years of clinical and research experience conducting qualitative research in cancer settings (JBR, LZ). Privacy of participants was ascertained at the start of every focus group/interview. All participants reported being in private settings at home, with no non-participants present. The PI (JBR) led all patient focus groups, with a co-author (LZ) acting as note-taker; either JBR or LZ led the partner interviews using the same standardized, structured interview guide, described in the next paragraph.

All focus groups and interviews used a semi-structured format following a pre-specified guide, which was designed to facilitate examination of the research questions. The same qualitative guide was used for patients and for partners. Development of the qualitative guide was informed both by conceptual models, including biopsychosocial models of sexual concerns in cancer (Bober and Sanchez Varela, 2012) and of coping with sexual concerns after cancer (Reese et al., 2010a), and by prior qualitative research conducted by our study team with a similar focus (e.g., Reese et al., 2016; Gorman et al., 2020; See Table 1 for examples of questions). The qualitative guide was thoroughly reviewed for appropriateness and completeness by the study team.

**Table 1:** Focus group guide.

*Part 1: Sexual and intimacy-related changes and coping efforts*	
1	[We know that the experience of metastatic breast cancer can affect women’s relationships with their partners in many ways]. How would you say your [your partner’s] breast cancer diagnosis and treatment has affected your relationship with your partner?
2	How about effects on your intimate relationship with your partner, meaning specifically your physical or sexual relationship with your partner? [Prompt for: sexual scripts changing, loss of sexual/intimate activities, roles changing toward caregiver/patient]
3	Many women report changes in their sexual enjoyment, interest, performance, or in their feelings about their body or their appearance. What have you noticed about how the breast cancer diagnosis or treatments have affected you [your partner] in these kinds of ways?
4	For women with recurrent disease: Some of you [your partners] may have recurrent disease, that is, you were [your partner was] diagnosed earlier and the cancer went away, and then it came back. I’m curious as to what you notice about how the experience is different this time versus previously in terms of its effects on your sex life or your intimate relationship?
5	Thinking about everything we have been talking about—including physical, emotional, and treatment effects on intimacy and sexuality—which of these changes have you found most challenging to deal with? Most bothersome?
6	Have you discussed these issues with your partner?
7	If discussed, when you and your partner have talked about these issues, how have these conversations gone?
8	Can you think of a time that you and your partner managed (or coped with) some of the physical or emotional challenges in your sexual relationship?
9	What kinds of things have you tried to help manage or cope with some of these challenges? How helpful were these in coping with these challenges?
10	Is there anything else that you can think of that would help you better manage (or cope with) some of these challenges?
11	How important is finding ways of coping with these challenges to you? To your relationship?
***Part 2: Information needs and intervention preferences***	
1	Thinking about everything we have just discussed, if we were to offer a program designed to address sexual and intimacy issues, what would you most hope to gain from this kind of program? What kind of information would you most want included?
2	If you were offered a program for you and your partner to help address sexual and intimacy issues like the ones you have mentioned today, what concerns would you have about participating?
3	What would make it easier for you to participate?
4	What concerns do you think your partner would have in participating in a program like this? What might help address these concerns?
5	What else would you like us to consider or have in mind as we put together this program? Anything that might make it likely you and your partner would participate?

All interviews began with the interviewer introducing herself (i.e., her role within Fox Chase Cancer Center and within the research project), reviewing the study purpose, and clarifying any participant questions. To facilitate rapport and transition the participants into a discussion of potentially more sensitive sexual concerns, the qualitative guide opened with a general relationship question, and from there, questions were broken into two main parts: (1) Sexual and Intimacy-Related Changes and Coping Efforts, and (2) Information Needs and Intervention Preferences. To encourage open communication of potentially sensitive topics, patients and partners were interviewed separately. Demographic data (i.e., age, race/ethnicity, education, employment status, and relationship duration) were obtained through online self-report questionnaires at the time of consent. Clinical data (i.e., diagnosis as either recurrent or as initial metastatic diagnosis and length of time since diagnosis) were obtained through coding transcripts and corroborated with patients’ charts when possible.

### Data Preparation

All focus group and interview discussions were audio-recorded and transcribed verbatim using the Zoom audio transcription feature. This feature functioned by automatically transcribing verbatim the audio of each recorded meeting and providing a text file that was divided into timestamped sections for each portion of recorded audio. These text files were then downloaded to a secure server and de-identified for any identifying information by one of the interviewers (LZ). The de-identified verbatim transcription was then checked and edited for accuracy by a research assistant through cross-referencing with the original audio recording and field notes taken during the interviews. Any sections of audio or transcribed text that were unclear were double-checked and clarified by one of the interviewers (LZ, JBR). Participants were not provided a copy of the interview transcripts.

### Data Analysis

Data were then analyzed following the steps outlined in the framework method (Gale et al., 2013, 2014), with regular meetings with the qualitative research expert on the study team (JG). The framework method is a systematic and pragmatic approach well suited to meet the objectives of the current investigation (Gale et al., 2013, 2014). The framework method provides a flexible, stepped approach to qualitative data management and guided our deductive (i.e., initial codes were developed based on the research questions) and inductive approach to analysis (i.e., codes were added to and modified as analysis progressed and ideas emerged from the data; Gale et al., 2013). The defining feature of the framework method is the matrix output, which is used to summarize and organize the qualitative data to facilitate analysis. In the matrix, the data are summarized according to transcripts and codes, which provides a structure for the data that allows for analysis by case (individual transcript) and code, and for comparison both within and across cases.

One of the lead coders (JBR) has substantial experience in conducting qualitative analysis using the framework method (Reese et al., 2016, 2017) as well as significant research and clinical knowledge on breast cancer and sexual wellbeing; all other coders completed training prior to embarking on coding. Training included readings and discussions on implementing the framework method and coding, including how to apply codes reliably, review of the codebook, and practice coding. At all stages in the coding process, discussions were held between coders to identify issues arising during the coding process and resolve any discrepancies in coding and uncertainties in applying codes or around code definitions.

Data from patients and partners were analyzed separately by four coders (JBR, LZ, KS, SM), and proceeded in several steps. First, two lead coders (JBR, LZ), constructed a coding book with definitions based on the research questions, the interview guide, and a close read of the first patient transcript, as described in the framework method. Because the same interview guide was used for both patients and partners, capturing their unique perspectives on the same issues, the codes were also the same with a few exceptions (e.g., a code called “infoneeds_patient” referred to partners’ perceptions of the patients’ information needs related to a sexuality intervention and only applied to partner transcripts.) The information gained directly from patients about this topic was more detailed and thus necessitated coding using more codes related to different areas of need. Second, once the codebook was finalized, two independent, trained coders (KS, SM) autonomously applied codes to the transcripts. Coders engaged in frequent discussion and reflection to discuss any emergent differences in coding application with the PI and, when needed, the qualitative expert (JG) until consensus was reached (Bradley et al., 2007). Finally, once codes were finalized, we then imported the data and coding into Dedoose to facilitate analysis and identification of themes. Specifically, we used the data from the code matrix in Dedoose to examine overlap of codes both within and across transcripts and compare codes across patients and partners for differences. Reviewing the data in the matrix is a key part of the framework method that leads to the identification of patterns within the data and thus themes. In identifying patterns, particular attention was paid to the most common codes and those that were farthest reaching (i.e., linked with a greater number of other codes), as this suggests important patterns in the data. From this examination, we derived key themes and subthemes that represented the qualitative data characterizing the experiences and coping efforts of patients and partners. In this step, relevance to the research questions, importance to participants, and novelty from a research perspective (e.g., distinctiveness from data observed in early stage breast cancer patients and partners) were strongly weighed. Regarding inter-rater agreement, for focus groups, the inter-rater agreement was high (92%) after reviewing data from one group, and as such further inter-rater agreement tests were deemed not necessary. All partner transcripts were double-coded and average inter-rater agreement was high (87%).

## Results

### Recruitment Data and Sample Characteristics

#### Patients

Twenty-one patients screened as eligible and were approached for participation; of these, 16 consented to the study (76%). Four patients who completed consent did not end up participating in the focus groups (3 were lost to follow-up during scheduling, and 1 reported discomfort in discussing her relationship). The total patient sample therefore included 12 women, 8 of whom were recruited through FCCC, with the remainder recruited through the community-based recruitment methods. Patient characteristics are provided in Table 2.

**Table 2:** Patient and partner characteristics.

Variable	Patients	Partners
	Mean (SD or range)	Mean (SD)
Age	50.2 years(11.2)	47.3 years(8.1)
Time since patient’s diagnosis	4.7 years (1 month-11 years)	n/a
	N (%)	N (%)
Race^*^		
*White*	11(91.7%)	5(83.3%)
*Black/African American*	1(8.3%)	1(16.7%)
Education		
*Bachelor’s degree or higher*	10(84%)	4(67%)
*Some college/associate’s degree*	2(16%)	2(33%)
Employment		
*Full-time*	4(33%)	5(83%)
*Part-time/self-employed*	2(17%)	1(17%)
*Retired*	3(25%)	0(0%)
*Full-time disability*	3(25%)	0(0%)
Length of relationship^**^		
*≤10 years*	2(17%)	3(50%)
*13–15 years*	3(25%)	1(17%)
*≥20 years*	7(58%)	2(33%)
Metastatic diagnosis status		
*Recurrent disease*	5(42%)	n/a
*New diagnosis*	7(58%)	n/a

* *All participants identified as having non-Hispanic ethnicity*.

** *All participants reported being married (vs. cohabitating) and having a household income of greater than $35,000*.

#### Partners

Six partners participated in study interviews; 1 consented but was lost to follow-up when attempting to schedule for the interview and 9 partners who the recruiter attempted to recruit opted out. In 5 cases, this occurred when the patient indicated that the partner would not be interested and so no further attempts were made to enroll the partner, and in 4 cases, this occurred when the patient indicated that she had spoken to the partner and the partner refused. Reasons for partner refusal included not being interested (*n* = 4), privacy/topic is sensitive or embarrassing (*n* = 3), or scheduling issues (*n* = 2). Partner characteristics are provided in Table 2.

### Results of Qualitative Analysis

#### Overview of Qualitative Findings

Five themes emerged from the analysis: (1) Patients’ Experiences of and Reactions to Sexual and Intimacy Changes and Concerns; (2) Partners’ Experiences and Roles; (3) Context of a Life-Limiting Illness; (4) Coping with MBC-Related Sexual Concerns; and (5) Information Needs and Intervention Preferences. Main themes, theme descriptions, corresponding subthemes, and illustrative quotes are shown in Table 3 (for themes 1 to 3) and Table 4 (for themes 4 and 5). For the purposes of verbatim quotations, we have provided fictive names for each of the participants. In this study, we defined saturation as when little additional information was gained from subsequent transcripts and used thematic coverage of higher-order groupings (themes) across transcripts in determining this (Hennink and Kaiser, 2022). Complete thematic coverage was achieved for the focus group data, offering support for achieving saturation. Coverage was high for partners as well, with only theme 3 appearing in fewer than all partner transcripts. With only 6 transcripts, full thematic coverage was not achieved for partners, but information obtained was robust (Malterud et al., 2016).

**Table 3:** Part 1: Qualitative themes 1–3 and illustrative quotes: patients’ and partners’ experiences of sex and intimacy after MBC diagnosis.

Main themes and subthemes	Illustrative quotes corresponding to themes
Theme 1: Patients’ Experiences of Sexual and Intimacy Concerns after Diagnosis of Metastatic Breast Cancer (MBC)— Significant disease burden amplifies sexual changes and challenges	
*MBC-related sexual changes and concerns*: Cumulative treatments and disease can amplify the severity and complexity of sexual problems, also may complicate responsiveness or ability to benefit from common treatment strategies	…all happened, you know a chemo-induced menopause and I had surgery, then, you know, ovaries out, double mastectomy. I said, like, really quick—at 45, I was saying, “well I’m one operation away from being a man” (Gloria, age 57). –
*Patients’ emotional reactions to sexual changes*: Emotional reactions to the sexual problems often include guilt and pressure, which can further exacerbate distress	…There are ways of being close without the intercourse, you know, without the goal…but again, I think it’s the, you know, you want to be a good partner and so it’s—I think a lot of that is, you know, the guilt part of it (Rose, age 67). –
*Combined effects of chronic physical and emotional sexual changes*: Combination of the persistent and often severe physical and emotional sexual concerns take a significant toll on patients and the relationship by wearing down hope and positive expectations, in snowball-like effect	It never goes away, you know, I mean it’s always like a mountain that you are trying to climb and find, you know, ways to, you know, overcome those obstacles (Kim, age 51). –
Theme 2: Partners’ Experiences and Roles— Partners’ experiences of sexual changes, emotional responses, and perceived impact of sexual changes play key role in shaping outcomes for the patient and the relationship	
*Awareness of MBC-related sexual changes*: Partners and patients describe a range of experiences and levels of awareness for partners of the changes in their sexual relationship, including the patients’ problems, their own sexual problems, and changes in the intimacy in their relationship	My husband thinks that’ll work – see, and I think you guys have same [issue] that I do. Men do not feel what we feel so they think that we are moist and we are not. And so I’m trying to say to him, “no it hurts” and he’s like, “I do not know what you are talking about. It’s not dry” and I’m saying; “yes, it really is dry” (Donna, age 39). –
*Partners’ emotional reactions to patients’ cancer-related sexual changes and concerns*: Partners describe substantial emotional effects of the patients’ sexual problems, including guilt at wanting to engage in sexual activity even when painful or not enjoyable for the patient	…the times that we have, you know, been—been intimate, it’s like…she’s not enjoying it [chuckle] and…I’m like, she’s doing it for my benefit, you know, and I feel bad that…like, that I’m making her do this…she’s, like, dry...and like, she’s tried different things, but, like, you know, it hurts... (Paul, age 43). –
*Partners’ roles in coping with sexual changes and concerns*: Partners’ experiences and emotional responses impact the intimacy in the relationship and on patients’ adjustment to their sexual problems (e.g., not wanting to cause pain leads to pulling back from intimacy whereas openness and patients in the partner can enhance the intimacy)	…I’ve found that when we have run into those periods in our relationship, then my husband kind of pulls back too, you know, for a while. And—and does not want to pressure me and does not want to initiate because he does not want to make me feel bad, you know. So, then—then that span of time grows and grows where nothing is—is happening (Kim, age 51). –
Theme 3: Context of a Life-Limiting Illness: Viewing the Future through the Lens of a Relationship with an “Expiration Date”— Living with life-limiting disease shapes the experience of sexual and intimacy changes, and responses to these changes	
*Viewing the relationship as time-limited*: The awareness of the life-limiting nature of the disease leads to changes in conceptualizing the relationship in general as a context for the intimacy	And now that I’m, like, deteriorating even further…it’s hard for him to watch this happen and it’s hard for me to watch him watch it happen…because, you know, we always thought we would, you know, retire together someday. It really seems unlikely that I’ll—I will get to a retirement age—ever, you know (Gloria, age 57). –
*Emotional consequences of life-limiting context for relationship*: There is a range of emotional responses and effects on intimacy, which for some couples included feeling closer, and for others, included additional stress changes in the definition of intimacy	…all these different stressors, you know, being able to provide financially, long term health insurance for me, you know, all these different factors play into just the additional stress on the relationship that did not exist before. And taking intimacy just even out of the equation (Kim, age 51). –
*Balancing life as normal with coping with MBC*: Patients and partners described a difficult balance between acknowledging the life-limiting nature of the disease versus living life as normal	Like, I always say, like, I’m—talk about it when it needs to be talked about but, like, especially, like, family-wise, like, with the kids and stuff, but then I sort of just put it on a shelf and it’s there. It’s always there and it can be taken down and looked at and talked about, but it does not need to be every day, and so we just sort of go on living as much as possible (Patricia, age 45). –

**Table 4:** Part 2: Qualitative themes 4–5 and illustrative quotes: coping with MBC-related sexual changes, information needs, and intervention preferences.

Main themes and subthemes	Illustrative quotes corresponding to themes
Theme 4: Coping with MBC-related Sexual Concerns— Coping efforts differed, seeming to bifurcate into effective and ineffective strategies; the stakes seem higher for poor coping given that the symptoms are worse	
*Effective approaches to coping with sexual concerns*: Couples who coped well and enjoyed intimacy despite sexual changes and limitations tended to approach the situation rather than avoid, tended to be flexible in their attitudes and behaviors toward sex and intimacy, acceptance without resignation, and open attitudes	It can be can be fun, you know, we can lube each other up. [laughter] So it’s, I do not know, I only see it as a positive honestly. I do not think there’s anything negative about it. (Robert, age 46) –
*Ineffective approaches to coping with sexual concerns*: Couples whose coping was less effective tended to employ cognitive and behavioral strategies that were often more rigid, or that suffered through the pain with a sense of duty or hopelessness, and that avoided the situation	And yes, it [sex] is very painful. Some days are better than others, you know even just trying to find different lubricants or different things that will help. Trying to find different methods and it’s very trying and taxing and sometimes I find myself just saying okay I’m going to do it regardless...Let us just hurry up and get through it…I’m sitting there like Jesus, help me, this is excruciatingly painful. But sometimes I just think, you know, what am I supposed to do? (Donna, age 39) –
*Role of communication in coping with sexual concerns:* Communication about sexual issues differed among couples; for some, communication was a way to connect and share changing needs or offer support, whereas for other couples, communication was strained initially and suffered further with MBC, and included significant avoidance and discomfort regarding discussing sex and intimacy	Our communication is very open, we talked about every aspect of it. He′s very supportive. Like I said, he’s a really good sport and he’s got a really good sense of humor so it all helps...Just to know that he is as supportive, as he is and just to know that he loves me no matter what [is helpful]. (Michelle, age 62) –
Theme 5: Information Needs and Intervention Preferences— Patients and partners reported needs for information on a range of issues and coping strategies, including the importance of addressing emotional aspects of intimacy and the relationship	
*Specific information needs*: Patients and partners commonly expressed a need for information on the effects of their treatments on their sexual function and intimacy, as well as for information on strategies to cope with their distressing sexual problems; some patients, however, who had lived for many years with sexual problems they felt were intractable, wondered at the potential usefulness of such information as they had tried a number of strategies that did not help	So I did not realize how much it was going to affect me until it started to just become very painful during intercourse. And my- the nurse did say to get the lubricant and I cannot use any estrogen and all of that stuff so that was a help. But I-I did not realize if I was supposed to be using it every day. (Brenda, age 46) –
*Beyond information: emotional needs*: A number of patients talked about the need for an intervention to address emotional issues and to normalize the sexual problems; for some patients, who had addressed their sexual issues, they felt they could benefit from help in addressing their fear of the other shoe dropping	I mean a program in that respect I think has to go even deeper than just sexual relations because there’s so many other psychological aspects to it that layer onto your ability to have that sexual relationship. Just because he’s-he has his own physical issues because of the psychological pressures, you know that he puts on himself… (Kim, age 51) –
*Motivation for participation in sexual concerns intervention*: Motivations differed, with some patients saying that the program would be worthwhile, and others reporting a lack of interest in participating in a program to address sexual intimacy, but the reasons diverged, ranging from a sense of pointlessness since the sexual problem was not perceived as addressable, to not prioritizing their issues, to not needing it since they had addressed it on their own, some said they thought their partners would not want to participate or might be more likely to discuss sexual issues with the physician than with a counselor	I do not think I have an issue to where I need to speak with a counselor or a specialist to talk about it or cope with it. Because I feel like I’ve been coping with it every day, and I’ve been coping with it for seven years. So, unless it was beneficial to where you guys had information that will really help me turn the situation around...I will feel like you know we are discussing it, but it’s really nothing that we can do about it. (Donna, age 39) –

#### Patients’ Experiences of and Reactions to Sexual and Intimacy Changes and Concerns (Theme 1)

##### MBC-Related Sexual Changes and Concerns

Within this theme, patients described a range of sexual concerns (e.g., physical, emotional/motivational, and relational), and they noted how the cumulative treatments they had received due to their advanced disease seemed to amplify the severity and complexity of their sexual problems and/or complicate their responsiveness to or ability to benefit from common treatment strategies. For instance, one woman described how, because she was “neutropenic all the time,” when she would engage in sexual intercourse with her husband, she would experience infections, saying, “you are in the hospital with sepsis. I’m way beyond, like, dilators and lubricants” (Gloria, age 57). The same patient spoke about how the extensive nature of her surgeries and treatments contributed to changes in her view of herself as a woman, saying, “Sometimes I’ll say something like, well … you know … chemo-induced menopause and I had surgery, then, you know, ovaries out, double mastectomy. I said—at 45, I was saying, ‘well I’m one operation away from being a man’” (Gloria, age 57). Another patient reported that “because of it being breast cancer, I could not take any hormones or estrogen or anything, so it threw me in a full-blown menopause with no help from anything and the situation went from bad to worse. You know, even now it’s [intercourse is] painful” (Donna, age 39). Loss of desire was also a significant problem, with one patient commenting on a total loss of desire for sex, saying, “For me, I’m just not like interested in, like, intimacy at all. I do not have like any urges or desire, like I could just be fine without it” (Joyce, age 33). Partners also commented on patients’ sexual concerns, including on the impact it had on the intimacy in their relationship. For example, one partner commented, “we have never been like a holding hand kind of couple … but, like, I feel like maybe I’d say we like would cuddle less because that usually like that comes before after or both, so I feel like there’s less there’s definitely less physical contact between us than before” (Paul, age 43).

##### Patients’ Emotional Reactions to Sexual Changes

The severity of the sexual problems experienced by many of the patients tended to be linked to substantial emotional reactions. For instance, the decreased ability to engage in sexual intimacy with their partners led to feelings of pressure and guilt, which served further to exacerbate patients’ distress about the sexual difficulties. Patients spoke of how their desire to be “a good partner” could bring with it pressure to engage in sexual activity and then guilt when the sexual experience does not go as planned (see quote in Table 3, Theme 1.b., Rose, age 67). In another example, one patient, who indicated that sexual intimacy with her husband was dictated by her faith beliefs, described the pressure she feels in needing to have sex with her husband despite pain, stating that “I just feel like it’s my job as his wife [to engage in intercourse despite pain] … and that coincides with my faith …” (Joyce, age 33); at a different point in the group, this patient also described guilt at not being able to engage in sexual intercourse despite this pressure, saying, “Sometimes I feel really bad … like, it’s not his fault I cannot do this [engage in intercourse].” Another patient noted that her inability to engage in sexual intercourse, compared to prior to cancer, caused emotional distress, saying that “… you are thinking, this is not how our sex life was going to be, you know, should be for such a young individual … so I put a lot of pressure on myself to perform, I guess, if you will” (Kim, age 51).

##### Combined Effects of Chronic Physical and Emotional Sexual Changes

This subtheme describes how the combination of the often severe, chronic physical sexual problems with the emotional reactions to these problems in the previous two subthemes takes a cumulative toll on patients and the relationship by compromising hope and positive expectations in a snowball-like effect. For instance, one patient noted that coping with sexual issues after metastatic breast cancer felt like a never-ending set of obstacles that was difficult to overcome (Kim, age 51; see Table 2, Theme 1.c); this same patient commented on the ups and downs she experienced in coping with these issues, stating that “it kind of went through peaks and valleys over the years since we have been dealing with it for so long.” When asked about the kinds of information she might benefit from, one patient commented on how the chronicity of her sexual problems made her skeptical that any information might help, saying, “I feel like I’ve been coping with it every day and I’ve been coping with it for 7 years. So, unless it was beneficial to where you guys had information that will really help me turn the situation around. I … feel like you know we are discussing it, but it’s really nothing that we can do about it [the sexual problem]” (Donna, age 39). One partner also described feelings of frustration and futility associated with attempting to engage in sexual intimacy in between treatments (when the patient might feel better physically) and expressed desperation his wife felt. Specifically, the partner commented, “Yeah, just so much going on and different periods, especially, but even the in between periods like, [there’s] not quite enough time to say things are back to normal. I do not know … between surgeries … when she is almost healed or things like that … here comes another surgery or here comes radiation … She’s even mentioned, like, ‘hey, maybe I can take some pain pills, and then we can go at it’ and I do not know if she was joking but that’s just a bad idea” (Raymond, age 42).

#### Partners’ Experiences and Roles (Theme 2)

##### Awareness of MBC-Related Sexual Changes

Both partners and patients described a range of experiences for partners in their sexual relationship, including partners’ own sexual difficulties (e.g., loss of interest in sex) as well as different levels of awareness of the patients’ sexual problems. Partners described their personal experiences with these sexual changes, their emotional responses, and the perceived impact of sexual changes on outcomes for the patient and the relationship. For instance, one partner commented on how his wife’s decreased sex drive had impacted his own, saying, “I feel like I do not have as much of, maybe, as a sex drive, as I used to either … it used to be like we’d go through, like those ruts and it’d be like we’d both be going like ‘We got to do that more often’ [laughter] you know … whereas it’s like now it happens and … I do not think it’s like ‘oh wow, got to do that again like tomorrow.’ It’s just less of a drive there … if that makes sense” (Paul, age 43). One patient commented on how the changes in their intimate relationship had impacted her husband’s sexual function, saying, “he has his own physical issues because of the psychological pressures, you know, that he puts on himself” (Kim, age 51). Occasionally, patients and partners commented on different levels of awareness partners had of the patients’ sexual changes, with one patient pointing out her husband’s lack of awareness of changes in her vaginal lubrication during sex (see quote, Table 3, Theme 2.a., Donna, age 39), and one partner noting an awareness of his wife’s loss of libido, stating, “it’s obvious when there’s interest and no interest and you are just doing it just to do it” (James, age 51).

##### Partners’ Emotional Reactions to Patients’ Cancer-Related Sexual Changes and Concerns

As with patients, partners also experienced emotional effects of patients’ sexual concerns and the changes in their intimate relationships, including guilt at wanting to engage in sexual activity even if it was uncomfortable for the patient (see quote in Table 3, Theme 2.a., Paul, age 43). Patients at times recognized partners’ conflicting feelings about engaging in sexual activity when not enjoyable for the patient. For instance, one patient stated, “So it’s just it’s a struggle, it really is … I mean, it’s stressful for him too because, obviously, he does not want me to be in pain. That’s not enjoyable” (Brenda, age 46). Another partner spoke of the cumulative effects of the patient’s pain during sexual activity combined with the pressure he felt to engage in sexual activity despite these problems, stating that “… it got—it got to a point recently where it [sex] wasn’t fun anymore … It’s that moment where … you got this time and you want to make the most of it … and it’s no longer fun and it’s stressful. That’s not a good spot to be in” (James, age 51).

##### Partners’ Roles in Coping With Sexual Changes and Concerns

When it came to coping, patients and partners described divergent ways of partners’ coping, with some participants describing avoidant behavioral reactions that tended to be less successful (i.e., led to ineffective solutions and did not improve outcomes) and others commenting on more approach-oriented reactions that tended to be more successful (i.e., led to effective solutions and better outcomes). For instance, as shown in Table 3, Theme 2.c, one patient (Kim, age 51) described how her husband tends to avoid initiating sexual intimacy because he does not want to pressure her, leading to an extended length of time in which no sexual contact is. In a similar vein, one partner described how neither he nor his wife takes the initiative to start sexual contact, leaving them without sexual activity, saying, “we do occasionally … snuggle … or spoon … you are thinking one thing and she’s thinking another thing, and you are on two different tracks and one veers this way and one veers that way, and then the moment is gone and it’s like alright goodnight” (James, age 51). By contrast, another patient observed, “my husband is very, like, patient and understanding. And it’s made us intimate in other ways, because I always used to think, like, sex was, like, intimacy but I’m learning that is not the case” (Mary, age 39). In a similar vein, one patient commented on how her husband’s positive view of her body helped her cope somewhat with her own negative thoughts about her body, saying, “he thinks I’m the sexiest thing that walked this earth … and I’m like, you know, it really helps my confidence because of the fact that he thinks that I’m beautiful and I’m sexy that I’m like, wow. I do not know what you see but okay” (Donna, age 39).

#### Context of a Life-Limiting Illness (Theme 3)

##### Viewing the Relationship as Time-Limited

This subtheme describes how the experience of living with a life-limiting disease shaped the experience of sexual and intimacy changes, and responses to these changes. First, patients tended to speak openly about how having metastatic disease reshaped how they viewed their relationship in general—which could serve as a backdrop for sex and intimacy—and their expectations for the relationship in the context of the life-limiting nature of their diagnosis. For instance, one woman described this sentiment by saying, “… you should not, I guess, view your relationship as having an expiration date” (Kim, age 51). Other patients concurred, including one woman who spoke about having to adjust to the idea that she may not live long enough to retire with her husband (quote shown in Table 3, Theme 3.a., Gloria, age 57). One partner described it similarly, stating, “When you get together with someone, you do not think about – oh, how’s this going to end, but when it says, “Oh, I have stage IV cancer … you see it – it’s like, real” (Thomas, age 62). Although the quotes in this and the other subthemes within Theme 3 are not specifically related to the sexual effects of the cancer, they help characterize the relationship context in which patients and partners are experiencing and coping with these sexual concerns, and we thus choose to include them.

##### Emotional Consequences of Life-Limiting Context for Relationship

This change in context to a time-limited relationship tended to come along with emotional and psychological impacts on the relationship. For instance, one patient commented on how difficult it was for her partner to adjust to their changed future, saying, “It’s hard for him [my husband] to watch this happen … we always thought that we would retire together someday” (Gloria, age 57). When asked about what should be included in an educational program addressing intimacy, the same patient who described her relationship as having an expiration date stated, “I mean a program, in that respect, I think, has to go even deeper than just sexual relations because there’s so many other psychological aspects to it that layer onto your ability to have that sexual relationship” (Kim, age 51). However, while some patients described stress on the relationship (see quote in Table 3, Theme 3.b.), other emotional responses were more positive or shifted over time. For instance, one woman acknowledged, “it’s been very stressful at times, but I think in—in the beginning, it brought us closer, to be honest with you, just because we were going through this together” (Brenda, age 46).

##### Balancing Life as Normal With Coping With Metastatic Cancer

The last subtheme within Theme 3 describes the challenges of trying to balance acknowledging the life-limiting nature of MBC while also wanting to live life as normally as possible. For instance, one patient described acknowledging that the terminal nature of her cancer is ever-present but that she chooses when to discuss it so that she can live a normal life without thinking of the issue constantly (Table 3, Theme 3.c., Patricia, age 45). One partner noted, “it’s just sometimes inconceivable the way your mind sometimes runs away on you and you got to kind of reel it back in quick” (James, age 51), illustrating his approach to coping with the constant presence of this diagnosis by “reeling back in” the negative thoughts. Another patient described using humor with her husband to attempt to strike this balance between an awareness of her terminal diagnosis, and experiencing life as normal: “we’ll be sitting at the kitchen table having something eat. … and I’ll say something like you know “the year after I die … one year after, I’m coming back and this kitchen better be spotless [laughter]” (Gloria, age 57).

#### Coping With MBC-Related Sexual Concerns (Theme 4)

##### Effective Approaches to Coping With Sexual Concerns

Couples who seemed to cope well and enjoy intimacy in their relationships despite the considerable sexual challenges tended to approach (rather than avoid) intimacy with a sense of openness, flexibility, and acceptance (without resignation). This often involved shifting toward a more inclusive definition of intimacy and toward non-intercourse sexual activity. For example, one patient shared, “My husband is awesome [chuckle] … he really tries. He brings home new toys and different things—like, anything that you can think about to help me. He's like, you know, ‘I do not want it to be one sided. I do not want to just be enjoying it by myself. I want you to enjoy yourself also. So whatever I can do, you know. Let us see if this works or how about we talk to your doctor about this’ …” (Donna, age 39). This open attitude and collaboration between patient and partner was viewed as critical by this couple for finding effective approaches. Another patient acknowledged the importance of “removing that psychological pressure so that you can kind of regain maybe that emotional intimacy between one another, where you can then start building those blocks toward, you know, the sexual relationship again” (Kim, age 51), illustrating the importance of addressing the emotional piece in order to regain physical intimacy. Another patient talked about how her physical intimacy had shifted from intercourse to other activities, saying, “And we do communicate and we do, you know—for me—I hate to say this, but, like, he rubs my head and to me that feels as good anything [laughter]. So, you know, there are ways of being close without the intercourse, you know, without the goal” (Rose, age 67).

##### Ineffective Approaches to Coping With Sexual Concerns

While some participants spoke of finding effective strategies for coping, others cited strategies that proved ineffective in that they left them without physical intimacy in their relationships. For instance, one patient, described earlier as having a religious faith proscribing sexual activity within marriage, noted, “I feel bad, like, it's not his fault that I cannot do this..so I do offer, you know, oral sex or something like that, but it's not also a preference …” (Joyce, age 33). Another patient described tolerating painful sex because the effort required to find sexual aids was “trying and taxing” (Table 4, Theme 4.b, Donna, age 39).

##### Role of Communication in Coping With Sexual Concerns

A key factor of both effective and ineffective coping strategies was often communication. For some participants, communication was a helpful way to connect and share changing needs or offer support. For instance, the patient whose partner helped her find sexual aids described open communication that facilitated success and intimacy in the process, stating, “We’re very open. Anything that we need to discuss and talk about, we do not shy away from it” (Donna, age 39). Additionally, one partner who described a resilient attitude toward sexual problems explained how he used communication to diffuse the effect of sexual problems within the relationship, saying, “I tell her all the time, I say ‘if this happens, it happens … if it does not, do not think that that's the defining moment of our relationship’” (Thomas, age 62). For others, communication was strained and included significant avoidance and discomfort regarding discussing sex and intimacy. For instance, one partner commented on the role of communication in avoidance of sexual activity, saying, “Yeah, like, I did not feel like, ‘hey, now’s the time, let us go, and she did not say, ‘I need it – give it to me,’ so it just did not happen (Raymond, age 42). Avoiding talking about the sexual relationship sometimes went along with the ineffective coping strategies described previously. For instance, one patient stated, “we do not talk about, like, it hurting ever, you know what I mean? I’ve explained it to him that it hurts, but now I just endure it, so um, yeah … That’s a choice of me, like, not deciding to tell him about it …” (Joyce, age 33).

#### Information Needs and Intervention Preferences (Theme 5)

##### Specific Information Needs

Patients and partners reported informational needs for a range of issues and coping strategies, including emotional aspects of intimacy and the relationship, effects of their treatments on their sexual function and intimacy, and strategies to cope with distress related to sexual problems. For instance, one partner wished he had received “basics of ‘here’s some things when you are on hormonal therapy that may be helpful during intimacy’” (Robert, age 46). Another partner who had lamented a loss of sex drive for his wife and himself stated that he would like “information on how she could get that drive back … and if there’s something I can do to help with that process …” (Paul, age 43). When asked what he most hoped to gain from an educational program, he explained, “a way for us to be more intimate and, I guess, for us and for her to want to be intimate, you know, like … to get that kind of connection back that we had … we still love each other, and we still like being around each other, so … I think it's more about, like, maybe getting back to that sex drive that's missing that way” (Paul, age 43).

##### Beyond Information: Emotional Needs

In addition to information about physical sexual side effects, some patients and partners discussed the importance of an intervention that could address emotional concerns pertaining to the sexual changes and more broadly, related to the relationship (see quote in Table 4, Theme 5.b., Kim, age 51). For instance, one partner commented on the importance of learning how to manage change in expectations for intimacy in light of the patient’s sexual function changes, saying, “I think it takes a little bit of willingness on everybody’s part to say, ‘although this is not exactly what we would like this to be, this is what it is, let us make—let us make this fun and it's certainly better than not having any intimacy at all … So managing, or you know, maybe you could have [a] part on managing your expectations” (Thomas, age 62). Several patients commented that hearing other women in the focus group discuss their sexual concerns was helpful to them because it let them know their experiences were common, thus normalizing the experience. For instance, one woman said, “So this, for me, is very educational and helpful because I’m not the only person going through this. There's lots of other people going through this and to me, this was very helpful for myself” (Brenda, age 46).

##### Motivation for Participation in Sexual Concerns Intervention

Many of the patients and partners described interest in learning new tools and strategies for coping with sexual concerns through the intervention, including learning specific information about sexual issues and ways for coping effectively. For instance, one patient commented, “If you guys are giving us tips on other ways to be intimate … That would be interesting” (Donna, age 39). Alternatively, some patients and partners described having already coped satisfactorily with sexual changes, such that an intervention would not be beneficial, as with one partner who stated, “I feel like we are pretty comfortable with where we are at with everything. I do not—I do not know, I mean, it sounds good …” (Robert, age 46). By contrast, others felt that their sexual concerns were so intractable as to be beyond hope. The chronicity of these issues seemed to play a role in these perceptions and lack of expectations for usefulness of interventions. For example, one patient said she would consider participating in a sexuality-focused intervention “to help someone else more than myself, because I almost feel like I’m a lost cause it’s—it’s I almost feel like it’s no hope at this point for me and it’s just I’m—I’m in this situation, or predicament and I just have to deal with the cards that have been dealt me” (Donna, age 39). Similarly, one partner stated, “So, I mean, I’ve been doing this for six years and she’s been keeping on top of it … for someone new, that [educational program] might be just the thing … we also have worked on our own selves … We’ve been continuing to grow through this so I do not know whether we need it anymore, but I’ll participate in whatever you present” (Thomas, age 62).

## Discussion

Overall, results of this qualitative investigation in patients with metastatic breast cancer (MBC) shed light on unique aspects of the experience of sexual concerns in this population, while also contributing to our understanding of both patients’ and partners’ perspectives on a changed view of the relationship itself in the context of a life-limiting diagnosis. In general, the types of issues patients and partners described were similar in nature to what has been reported in the literature for women with non-metastatic breast cancer (e.g., Rosenberg et al., 2014). However, the substantial disease and treatment burden associated with MBC seemed to heighten the acuity of the sexual problems that women with MBC experienced and potentially further compromise the effectiveness of coping options. For instance, some patients with MBC spoke of vaginal dryness so severe that engaging in sexual intercourse could cause a potentially dangerous infection as the vaginal tissue would likely tear during the activity; others spoke of having tried numerous sexual or vaginal aids without relief such that they had lost hope in ever engaging in comfortable sex. The heightened severity of these concerns seemed to increase the risk for considerable adverse emotional and interpersonal consequences for patients (e.g., guilt over not being able to engage in intercourse) and partners alike (e.g., guilt in pressuring the patient to engage in sexual activity despite pain/discomfort). In sum, while these findings echo the emotional consequences of sexual concerns seen for patients with breast cancer generally, they also suggest that these experiences may be especially pronounced for some women facing metastatic disease.

This study provided novel findings on MBC partners’ perspectives of cancer-related sexual changes and concerns, with the role of partners being especially notable in discussions of coping with the sexual concerns. For example, partners’ involvement helped to determine the directions and effectiveness of strategies patients described in coping with sexual concerns, including whether to approach or avoid the sexual issues in the relationship. For instance, partners’ openness to find solutions for vaginal symptoms that were interfering with sex seemed to help allay patients’ negative feelings associated with their sexual concerns while also leading to creative solutions. By contrast, partners who seemed to cope by avoiding discussing the topic or by withdrawing from physical intimacy inadvertently perpetuated a lack of intimacy and feelings of disconnection between the couple. In sum, these findings are consistent with those of prior studies demonstrating the important role of the partner in BC-related sexual concerns (Ganz et al., 2002; Fobair and Spiegel, 2009).

With these findings in mind, a couple-based intervention approach seems well suited to addressing sexual concerns because it addresses partners’ coping and the interpersonal context of such concerns. Indeed, prior studies provide convincing evidence for couple-based interventions in addressing women’s sexual concerns after cancer (Taylor et al., 2011). However, it is also worth noting that sexual health interventions delivered to women on their own also have demonstrated efficacy (Hummel et al., 2017b; Bober et al., 2018). Given that recruiting partners to a couples’ only intervention study can add to the challenges inherent in recruiting to these studies (Regan et al., 2013), there is value in considering multiple types of interventions depending on patients’ and partners’ needs and preferences. Moreover, findings from our study suggest that patients and partners who have experienced perceived failure in trying to address their sexual problems may doubt the potential benefits or effectiveness of a proposed intervention. A belief such as this (that sexual problems are beyond hope), could pose further challenges to researchers and others interested in offering interventions to patients and partners in this population, while also suggesting that earlier intervention—given before such feelings of hopelessness and futility can pervade—may have the best chance for uptake and possibly effectiveness in this population.

Novel findings from this study include ways that the life-threatening nature of MBC seemed to shape patients’ and partners’ perspectives on the relationship. Most strikingly, some patients openly described their relationship in terms that indicated an endpoint (i.e., “as having an expiration date”). In the same vein, some patients acknowledged competing needs for confronting the reality of the diagnosis with still maintaining normalcy in the relationship. These ideas echo those found in prior research that suggest that the stress of the life-limiting diagnosis itself can influence women’s intimate relationships and sexuality through stress and emotional distress (Vilhauer, 2008), above and beyond physical side effects. Further, although the quotes supporting these ideas were often not specifically related to sexual concerns, they are relevant to the focus of the present investigation because (a) they help us understand the context in which patients and partners are experiencing and coping with these sexual concerns (i.e., the partnered relationship), and (b) they have implications for interventions and clinical practice.

A major implication of these findings is the importance of addressing the broader context of survivors’ and partners’ emotional and interpersonal needs during discussions of sexual concerns related to the cancer. Researchers aiming to design an intervention addressing sexual concerns in this population could consider building off prior interventions that have aimed to help women and partners cope with the diagnosis of advanced breast cancer (e.g., Northouse et al., 2005). Specifically, researchers could consider including information on managing feelings and expectations about the relationship in the context of the metastatic diagnosis alongside information on common sexual problems and coping strategies. Similarly, clinical discussions of cancer-related sexual changes could also allow for discussions of changes in expectations or priorities around sex and intimacy within the relationship. For example, when raising the issue clinically, clinicians could acknowledge this context explicitly (e.g., “I know that you have a lot to handle right now, and this may not be top of your mind, but sexual concerns can come up for many women who have this diagnosis”). The broader context should most likely be acknowledged throughout an intervention or clinical process (e.g., therapy) as patients’ and partners’ thoughts and feelings on the subject may change over time. Overall, these findings are consistent with prior research demonstrating a range of types of changes experienced in the relationships of women with metastatic breast cancer (Valente et al., 2021) and highlight the importance of considering the context of the metastatic diagnosis and the changed overall relationship when addressing sexual concerns for partnered women with MBC.

### Clinical Implications

In addition to implications for intervention development, this study has several important clinical implications. First, when inquiring about sexual concerns, clinicians may benefit from awareness of the severity of the common sexual concerns in the context of the heavy disease and treatment burden. Having appropriate referrals is important when treating patients who are experiencing severe or intractable sexual problems, or significant emotional concerns or relationship distress alongside sexual concerns. In addition, educating clinicians to broach the topic of sexual health could normalize the conversation in clinical settings, decrease perceived stigma associated with discussing sexual issues, and hopefully facilitate productive dialogue between clinicians and their patients in the future (Bober et al., 2016; Reese et al., 2017). Second, given the important role of partners in coping with sexual concerns, clinicians should ideally provide partners with information about potential sexual side effects along with the patients and, if it aligns with patients’ wishes, should include partners in discussions of sexual concerns. Including the partner in such discussions is also consistent with clinical guidelines for discussing sexual health and function after cancer (Carter et al., 2017). Our findings suggest that including partners in discussions of sexual concerns, when desired by the patient, could help alleviate some burden from patients by confirming that the sexual concerns are common and related to the treatments, while also setting a stage for joint coping efforts. Finally, many of the participants who described coping effectively displayed signs of coping flexibility, a concept that can be useful in guiding coping efforts with cancer-related sexual concerns (Reese et al., 2010a). This concept involves making shifts in thoughts and behaviors related to how sexual activity and function are defined and enacted within the relationship. Clinicians working with patients after cancer might be encouraged to notice potentially rigid or inflexible beliefs when discussing breast cancer-related sexual issues (e.g., “I do not want to use artificial lubricant during sex because that feels weird”). They could then validate the perspective while encouraging greater flexibility (e.g., “I can understand how this might feel different from what you are used to, but it could be worth a shot, as they are safe from a cancer standpoint and many women and their partners find them very helpful in getting back to sexual activity. What are your thoughts about that?”). Clinicians could also encourage a sense of openness and curiosity toward new coping strategies, such as using a vibrator during sex, spending more time in non-intercourse sexual activities, pursuing pelvic floor physical therapy, or other strategies that patients and partners may not have previously considered but that could prove helpful. With that said, we acknowledge that there are significant barriers to open patient-provider communication about breast cancer-related sexual health concerns (McClelland et al., 2015; Reese et al., 2017). Many clinicians could thus benefit from obtaining further training to feel comfortable enough to raise such discussions effectively with their patients.

### Limitations

There are several important limitations to this research study to acknowledge. First, although we made efforts to recruit using both site-based and community-based methods in the hopes of recruiting a more diverse sample, the sample was ultimately limited in its racial and ethnic diversity as well as sexual orientation and gender identity. Some research suggests that the types of sexual concerns and information needs that breast cancer patients report tend to be similar across racial and ethnic backgrounds (Reese et al., 2020a), although some differences in experiences and preferences regarding communication about sexual issues have been found (Anderson et al., 2021). Nevertheless, more research is needed specifically examining sexual health and communication in women with breast cancer of diverse backgrounds and identities, especially those with MBC. Second, the fact that women had to be partnered in order to participate may limit generalizability of findings to women with MBC who experience sexual concerns but are unpartnered, and may also contributed to limitations in diversity. Third, the sample size was small, particularly for partners. Indeed, although we had initially hoped to obtain similar participation from patients and partners in the study, challenges in recruiting partners compromised our ability to meet the initial objective, leading to more limited participation by partners in the study, with most partners who opted not to participate ending up not participating after their partners (i.e., the patients) refused on their behalf. As well, several partners who refused study screening reported discomfort in discussing issues of sex and intimacy. Indeed, the challenge in recruiting partners to couple-based studies, which generally requires the agreement of both patients and partners, has been reported in studies in breast and other cancers (Fredman et al., 2009; Reese et al., 2018); this is especially true for late-stage disease (Regan et al., 2013). Further, when recruitment of partners to breast cancer studies is optional, sample sizes of partners tend to be smaller than those of patients in the same studies (Giese-Davis et al., 2000; Hummel et al., 2017a). Thematic coverage in our patient data supported the notion that saturation was achieved; reaching saturation with a small number of focus groups can be possible in less heterogeneous samples, as ours is (Hennink and Kaiser, 2022). Although complete thematic saturation was not achieved for partners, we observed substantial overlap in the perspectives across partners as well as complementary findings when compared to patients’ own perspectives, lending support for the robustness and important contributions of the data obtained from partners. Finally, the sample was too small to explore how clinical factors like length of treatment for metastatic disease, length of time since diagnosis, status of having recurrent versus primary metastatic diagnosis, or location of metastasis might impact findings. Future studies on intimacy using larger sample sizes of partners of patients with metastatic breast cancer could add to the findings of the present investigation.

### Conclusion

Findings from the present investigation contribute to our understanding of challenges to sex and intimate relationships for patients living with MBC. An important finding is that the often-heightened severity of sexual concerns for patients with MBC, compounded by the terminal nature of the disease, may place patients and partners at risk for potentially significant adverse emotional and interpersonal consequences. Results suggest that when developing interventions, researchers should consider acknowledging and potentially addressing the range of emotional responses to sexual concerns, helping patients and partners navigate changes in their intimate relationships, and equipping both patients and partners with skills for coping with these changes amidst the considerable physical and emotional burden of MBC. Addressing unmet sexual health and intimacy needs is a fundamental part of whole-person cancer care that has important implications for the wellbeing and quality of life of patients with MBC and of their partners.

## Data Availability Statement

The datasets presented in this article are not readily available because of the sensitive nature of the qualitative data in order protect patient confidentiality. Requests to access the datasets should be directed to jennifer.reese@fccc.edu.

## Ethics Statement

The studies involving human participants were reviewed and approved by Fox Chase Cancer Center Institutional Review Board. The participants provided their written informed consent to participate in this study.

## Author Contributions

JR, LZ, AZ, LP, MD, and JG contributed to conception and design of the study. JR, LZ, SM, KS, LP, AZ, and BC contributed to resources acquisition and data collection. JR, LZ, SM, KS, and JG conducted analyses. JR wrote the first draft of the manuscript. LZ contributed significantly to a revised next draft. All authors contributed to the article and approved the submitted version.

## Funding

This study was funded in part by 134607-RSG-029-01-CPPB from the American Cancer Society to JR, PhD and P30 CA006927 funded by the National Cancer Institute of the National Institutes of Health. The funding agencies had no role in the conduct of this study.

## Conflict of Interest

The authors declare that the research was conducted in the absence of any commercial or financial relationships that could be construed as a potential conflict of interest.

## Publisher’s Note

All claims expressed in this article are solely those of the authors and do not necessarily represent those of their affiliated organizations, or those of the publisher, the editors and the reviewers. Any product that may be evaluated in this article, or claim that may be made by its manufacturer, is not guaranteed or endorsed by the publisher.
